# Association between the dietary inflammatory index and infertility: a systematic review and meta-analysis

**DOI:** 10.3389/fnut.2025.1599782

**Published:** 2025-08-26

**Authors:** Yasamin Zahedi, Shayan Bonyanpour, Seyed Danial Alizadeh, Sarah Ravankhah, Alireza Zare, Bahareh Izadi, Shant Apelian, Eghbal Sekhavati, Reza Tabrizi

**Affiliations:** ^1^Student Research Committee, Fasa University of Medical Sciences, Fasa, Iran; ^2^Sina Trauma and Surgery Research Centre, Tehran University of Medical Sciences, Tehran, Iran; ^3^Yasuj University of Medical Sciences, Yasuj, Iran; ^4^Shiraz University of Medical Sciences, Shiraz, Iran; ^5^Health Policy Research Center, Institute of Health, Shiraz University of Medical Sciences, Shiraz, Iran; ^6^Department of Obstetrics, Gynecology and Reproductive Sciences, Yale School of Medicine, New Haven, CT, United States; ^7^Noncommunicable Diseases Research Center, Fasa University of Medical Sciences, Fasa, Iran; ^8^Clinical Research Development Unit, Valiasr Hospital, Fasa University of Medical Sciences, Fasa, Iran

**Keywords:** infertility, immunonutrition diet, dietary inflammatory index, inflammation, diet

## Abstract

**Introduction:**

To evaluate the association between the dietary inflammatory index (DII) and risk of infertility in human participants.

**Methods:**

A comprehensive search was conducted using the Scopus, PubMed, Web of Science, Cochrane, Embase, and Google Scholar databases until January 2025. All observational studies that reported an association between the DII and infertility in human participants were included. Data were extracted on study characteristics, DII scores, and infertility outcomes. Meta-analyses were conducted using random-effects models, and the heterogeneity of studies was evaluated using I^2^ statistics.

**Results:**

Of the 801 studies screened, nine met the inclusion criteria, involving 17,711 individuals. The analysis revealed a significant association between a pro-inflammatory diet (characterized by high DII scores) and infertility (odds ratio (OR): 1.61, 95% CI: 1.32–1.95) among participants in the highest quartile of the DII. Additionally, each unit increase in the DII was associated with a 10% higher risk of infertility (OR, 1.10; 95% CI: 1.05–1.15). Subgroup analyses revealed a significant association between anti-inflammatory diets (low DII scores) and infertility in men and increased infertility risk in both the Iranian and US populations and in female participants on a pro-inflammatory diet. The sensitivity analysis indicated that the overall OR remained stable, with the results not being significantly influenced by the exclusion of individual studies.

**Conclusion:**

These findings highlight the potential role of inflammation-related dietary factors in reproductive health and suggest that dietary modifications targeting inflammation could be a promising intervention for infertility management. Further randomized controlled trials are needed to confirm these findings and to establish causal relationships.

**Systematic review registration:**

This systematic review is registered in PROSPERO with code: CRD42024567145. URL: https://www.crd.york.ac.uk/PROSPERO/view/CRD42024567145.

## 1 Introduction

Infertility refers to the failure of a couple to conceive after 12 months of frequent unprotected sexual activity (three to four times a week) for women under 35 years and after six months for women over 35 years (1). According to epidemiological reports, the incidence of infertility in developed countries, reporting rates of 3.5–16.7%, compared to 6.9–9.3% in developing countries, makes infertility a major health concern (2). Studies indicate that female and male infertility increase by 0.37 and 0.29% yearly, respectively (3). Male factor infertility accounts for 20–70% of all infertility cases (4). Although there are methods to treat infertility, identifying adjustable factors and non-pharmaceutical therapies may be effective in improving fertility outcomes (5–7). In addition, nutrition is an important lifestyle factor that crucially influences fertility-related outcomes (8, 9).

Improving dietary habits before pregnancy and adhering to the Food-Based Dietary Guidelines can significantly impact fertility outcomes (10, 11). This guideline recommends eating fewer foods high in saturated fat and free sugars and eating more foods containing substantial amounts of unsaturated lipids and nutrients (11). Although the precise mechanism underlying the diet’s effect on fertility is still unknown, inflammation seems to be one of the main factors. Combinations of various nutrients found in diets can have complex interactions with each other and ultimately affect inflammatory status and subsequent health outcomes (12). Chronic inflammation can harm fertility, leading to endometriosis, irregular menstrual cycles, implantation failure, and frequent miscarriages (13). For example, anti-inflammatory components, specifically omega-3 fatty acids such as eicosapentaenoic acid and docosahexaenoic acid, influence menstrual disturbances such as dysmenorrhea by decreasing the levels of prostaglandins in the blood (14, 15). Additionally, inflammation associated with endometriosis hinders decidualization, where the endometrium undergoes changes in preparation for pregnancy, diminishes progesterone levels, a sex steroid known for its anti-inflammatory effects, and disrupts the endometrial lining (16). Studies have shown that the addition of vitamins E and C can reduce indicators of inflammation and oxidative stress in women with endometriosis (17). Supplementing with zinc, selenium, omega-3 fatty acids, and coenzyme Q10 notably enhanced sperm concentration and motility, while omega-3 fatty acids and coenzyme Q10 also led to an increase in the total sperm count (18, 19). In contrast, diets that consisted of meat, potatoes, full-fat dairy, coffee, alcohol, and sugar-sweetened drinks have repeatedly been linked to reduced sperm quality and fertility (20). Overall, nutritional therapies that mitigate inflammation in both males and females before conception may improve pregnancy outcomes and reduce the necessity for additional workups (21).

The dietary inflammatory index (DII) is an emerging method designed for evaluating the inflammatory potential of diets and is based on 45 food parameters with either pro-inflammatory or anti-inflammatory effects. DII is associated with levels of interleukin 2 (IL-2), interleukin-1β (IL-1β), interleukin 1–6 (IL-6), tumor necrosis factor (TNF), and C-reactive protein (CRP) (22). Recently, several studies have assessed the impact of the DII on various diseases, such as many types of cancers, cardiovascular diseases, metabolic syndrome, diabetes, and mental health (23–26). Furthermore, numerous studies have explored the relationship between infertility and the DII, many of which reported a positive relationship, and some studies, such as those by Fang-Hua Liu et al., which were conducted on the DII and the risk of asthenozoospermia—a major pathological indicator of male infertility—found no significant negative relationship (27–35).

According to the aforementioned cases, the increasing prevalence of infertility and the high psychological and financial costs it imposes on families and countries, and the conflicting results of the studies mentioned above, indicate that investigating the association between the DII and infertility can help us better treat and prevent this problem. The results of this study can lead to a reduction in the prevalence and prevention of infertility, reduce financial costs and psychological burden, and provide a more complete view of the relationship between the DII and infertility. Despite several studies on the association between infertility and the DII, no previous meta-analysis has evaluated this subject. This meta-analysis was performed for the first time to summarize the existing evidence and determine the potential link between the DII and the odds of infertility.

## 2 Materials and methods

### 2.1 Search strategy

Reporting adhered to the Preferred Reporting Items for Systematic Reviews and Meta-Analyses (PRISMA) 2020 checklist (36). This systematic review is registered in PROSPERO with code: CRD42024567145. A systematic search was conducted using Scopus, Web of Science, PubMed, Cochrane, Embase, and Google Scholar from their inception until January 2025. The search strategy for finding relevant studies was as follows: (“Dietary inflammatory index” or DII or “Inflammatory diet” or “Inflammatory diets” or “Anti-inflammatory diet” or “Anti-inflammatory diets” or “Pro-inflammatory diet” or “Pro-inflammatory diets” or “Dietary score” or “Dietary scores” or “inflammatory potential of diet” or “dietary inflammation potential”) and (Infertility or Sterility or Subfertility or Sub-Fertility or “Reproductive Sterility” or “Female Infertility” or “Postpartum Sterility” or “Female Sterility” or “Female Subfertility” or “Female Sub-Fertility” or “Male Infertility” or “Male Sterility” or “Male Subfertility” or “Male Sub-Fertility “or Reproduction or “Human Reproductive Indexes” or “Human Reproductive Index” or “Human Reproductive Indices” or “Reproductive Period” or “Reproductive Periods”).

### 2.2 Eligibility criteria

All observational studies with cohort, case–control, or cross-sectional designs that reported an association between the DII and male or female infertility in human participants were included.

The exclusion criteria were (1) unavailable or insufficient data; (2) animal studies; (3) publications in languages other than English; (4) letters to the editor; conference abstracts without a full article, case series, or case reports; (5) studies that did not use a DII score; (6) studies that did not investigate infertility; (7) studies that did not report the association between the DII and infertility; and (8) studies for which the full text was not available.

### 2.3 Data collection and quality assessment

Following deduplication, two independent authors evaluated each title or abstract. Disagreements were addressed through group discussions and adjudicated by an additional reviewer. The authors subsequently examined full-text articles that adhered to the predefined inclusion parameters. Then, eligible data from the included articles were collected. The collected information included the first author, study location, year of publication, sample size, diet assessment tool, and features of the case and control groups, such as mean age, adjusted odds ratio (OR), and body mass index.

Two authors, YZ and SH-B, independently evaluated the study quality using the Newcastle-Ottawa scale for cross-sectional and case–control studies (37). This tool assesses three primary domains: selection, comparability, and outcome. Studies were characterized according to their total scores: scores of 3 or lower were considered poor quality, scores of 4 to 6 indicated fair quality, and scores of 7 or higher were classified as good quality.

### 2.4 Statistical analysis

Across original research examining the DII and infertility relationships, individuals were grouped into four quartiles according to their DII scores, with a higher DII indicating a pro-inflammatory diet and a lower DII reflecting an anti-inflammatory diet. Then, meta-analyses were conducted on each quartile to compare the odds of infertility across the quartiles, with Q4 showing higher odds compared to Q1.

In addition to analyzing the data based on DII quartiles, the meta-analysis included studies that reported DII scores as continuous variables. These studies assessed the effect of a unit increase in the DII on the odds of infertility, allowing for the evaluation of incremental changes in risk. Only studies with continuous DII data were used to calculate the ORs for each unit increase in the DII.

The meta-analyses were performed using STATA version 14 (Stata Corp., College Station, TX, United States). The results are presented as OR with 95% confidence intervals (CI). A random-effects model with the DerSimonian-Laird method was applied to combine the effect sizes. Inter-study heterogeneity was assessed using the I^2^ statistic. If the I^2^ value exceeded 50% or the *p*-value was less than 0.1, the data were considered heterogeneous. Additionally, to investigate heterogeneity sources, both subgroup and sensitivity analyses, which assess the impact of excluding specific studies on the overall effect size, were performed. Publication bias was assessed using two complementary approaches: funnel plots and Egger’s test.

## 3 Results

**Figure 1:**
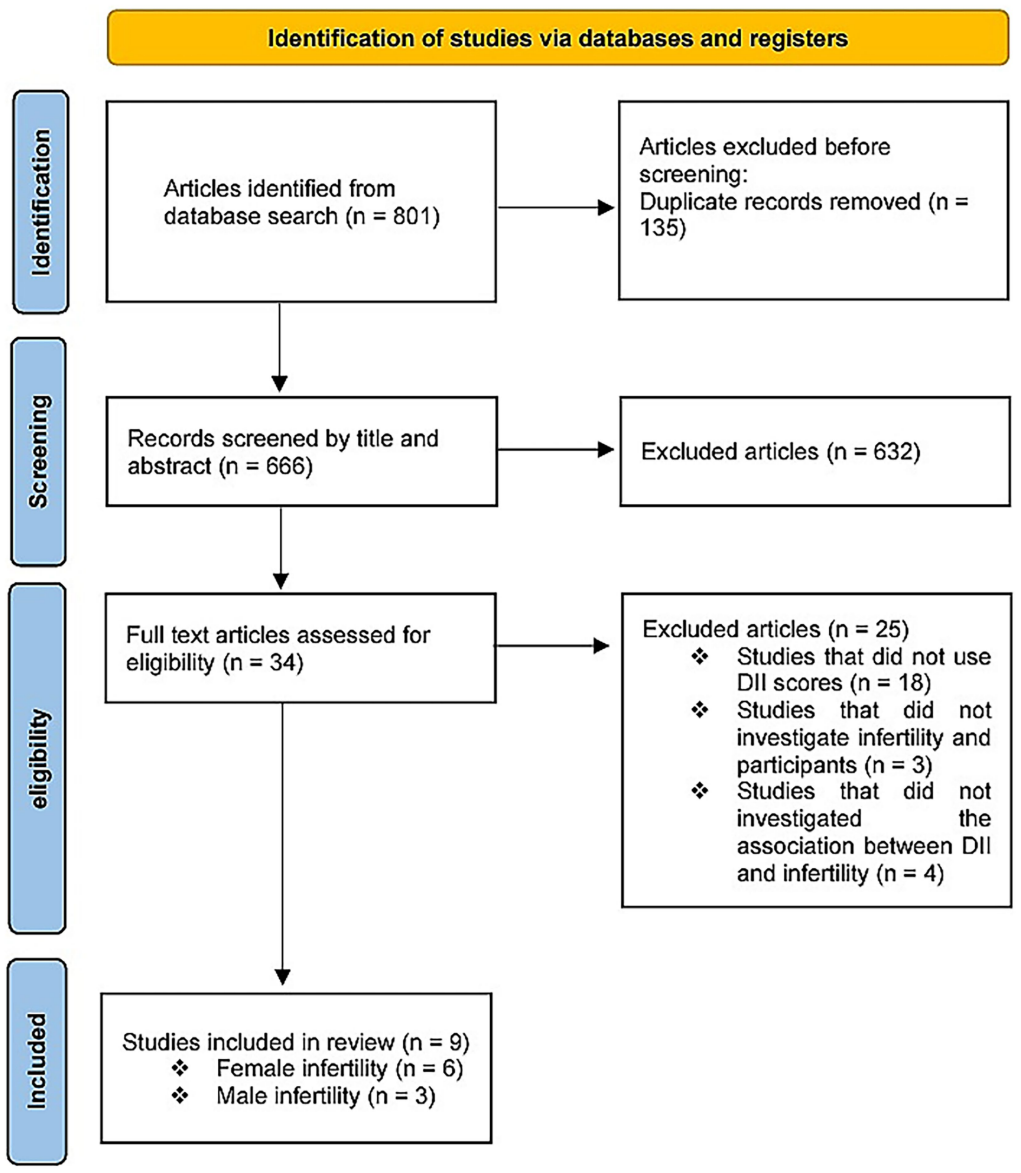
PRISMA flowchart illustrating the study selection process.

The database search identified 801 studies. A total of 135 records were excluded due to duplication, and 632 records were excluded from title and abstract screening. Of the remaining 34 publications, 25 were excluded. Nine studies were deemed eligible following a full-text review. Among these, six studies (27, 30–32, 34, 35) evaluated the DII and female infertility, and three studies (28, 29, 33) assessed male infertility (Figure 1).

Overall, 17,711 individuals were included in this meta-analysis, of whom 2,830 were infertile. Studies were conducted in three countries: the United States, China, and Iran. Five studies used the Food Frequency Questionnaire to assess and calculate an individual’s DII, three studies collected their data by conducting a recall interview at a Mobile Examination Center, and the other used a self-reported questionnaire. The main characteristics of each study with adjusted ORs are summarized in Tables 1, 2, which show the results of the risk-of-bias assessment.

**Table 1:** Main characteristics of each study.

Study	Country	No.	Sex	Adj. OR [95% CI]	Diet assessment tool
Aghaei et al., 2023 (27)	Iran	600	Female	1.70 [0.97–2.95]	FFQ
Dabagh et al., 2023 (28)	Iran	210	Male	2.93 [1.18–7.24]	FFQ
Liu et al., 2021 (29)	China	1,130	Male	0.86 [0.58–1.27]	FFQ
Lu et al., 2024 (30)	USA	2066	Female	1.78 [1.03–3.11]	Self-reported questionnaire
Moludi et al., 2023 (31)	Iran	4,437	Female	1.76 [1.57–2.02]	FFQ
Qi et al., 2024 (32)	USA	3,496	Female	1.59 [1.03–2.45]	MEC and a telephone interview
Taheri Madah et al., 2023 (33)	Iran	88	Male	1.83 [0.55–6.31]	FFQ
Wang et al., 2024 (34)	USA	3,071	Female	1.71 [1.17–2.51]	MEC and a telephone interview
Xu et al., 2024 (35)	USA	2,613	Female	1.61 [1.05–2.47]	MEC and the second interview’s information are collected by telephone 3–10 days later.

All studies were designed cross-sectionally, except for Aghaei et al. and Liu et al., which were case–control studies. 95% CI, 95% confidence interval; FFQ, Food Frequency Questionnaire; MEC, Mobile Examination Center; OR, odds ratio.

**Table 2:** Quality assessment results using the Newcastle-Ottawa Scale.

Cross-sectional studies^*^	1	2	3	4	5	6	7	Quality score
Dabagh et al., 2023 (28)	1	1	0	2	1	1	1	Good
Lu et al., 2024 (30)	1	1	1	2	1	1	1	Good
Moludi et al., 2023 (31)	1	1	1	2	1	1	1	Good
Qi et al., 2024 (32)	1	1	1	2	1	1	1	Good
Taheri Madah et al., 2023 (33)	1	1	0	1	1	1	1	Fair
Wang et al., 2024 (34)	1	1	1	2	1	1	1	Good
Xu et al., 2024 (35)	1	1	1	2	1	1	1	Good

Case–control studies^**^	1	2	3	4	5	6	7	8	Quality score
Aghaei et al., 2023 (27)	1	1	1	1	2	2	1	1	Good
Liu et al., 2021 (29)	1	0	0	1	2	2	1	1	Good

^*^(1) Is the sample truly representative of all cases? (2) Is the sample size satisfactory? (3) Is the response rate satisfactory? (4) Ascertainment of exposure using a validated measurement tool or a non-validated screening/surveillance tool, but the tool is available or described. (5) Did the study investigate potential confounders? (6) Were the outcomes assessed by independent blind assessment, record linkage, or self-report? (7) The statistical test used to analyze the data was clearly described and appropriate. ^**^(1) Was the case definition with independent validation? (2) Were the cases representative of their populations? (3) Was the control group selected from the community? (4) The control group did not have any history of this outcome. (5) Did the study control for age, or did it control for additional factor(s)? (6) Was the ascertainment of exposure done by secure record or structured interview, where it was blind to the case/control status? (7) Was the method of ascertainment the same for cases and controls? (8) Was the non-response rate the same for both case and control groups?

The findings of the meta-analysis regarding the effect of the DII on infertility among participants adhering to an inflammatory diet in comparison with the first quartile are explained as follows:

### 3.1 Second quartile

Nine studies were evaluated to examine the DII effects on infertility among individuals in the second quartile diagnosed with infertility. One of the studies failed to assess the relationship between the first and fourth quartiles. Therefore, they were excluded from this study. Ultimately, eight studies assessed the relationship between quartiles one and four.

**Figure 2:**
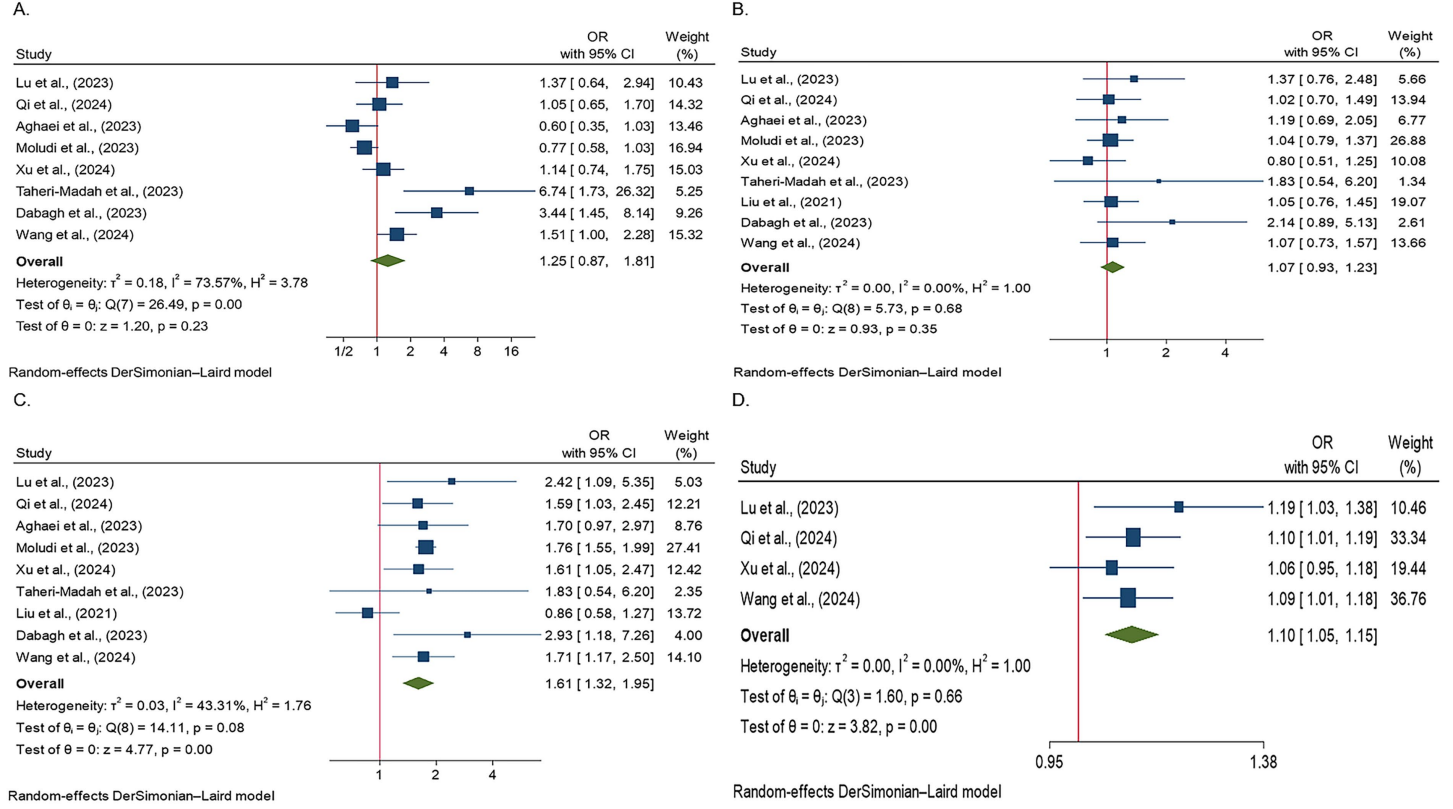
Forest plots illustrating the odds of infertility across different DII score quartiles. The figure presents the ORs with 95% CIs for each study and overall pooled estimates. Studies were grouped according to the DII quartiles, with the odds of infertility displayed for each quartile as follows: **(A)** Forest plot showing the odds of infertility in the second DII quartile. The effects of the DII on infertility in this group were not statistically significant; **(B)** Forest plot showing the odds of infertility in the third DII quartile. No statistically significant effects of the DII on infertility were observed in this group; **(C)** Forest plot showing the odds of infertility in the fourth DII quartile. The results were statistically significant, indicating that individuals adhering to a pro-inflammatory diet had 61% higher odds of infertility compared to those with lower DII scores; and **(D)** a forest plot showing the odds of infertility in studies using continuous DII data. A positive association between the DII and infertility prevalence was observed, with each unit increase in the DII associated with a 1.1-fold increase in the risk of infertility.

**Figure 3:**
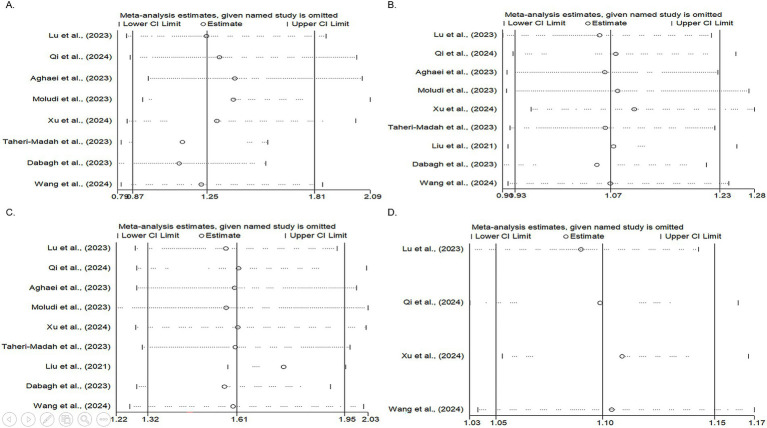
Forest plots illustrating the results of the sensitivity analysis (leave-one-out meta-analysis) for the second, third, and fourth DII score quartiles, as well as continuous DII data. The plots display the ORs with 95% CIs for each study and the overall pooled estimated OR. **(A)** Sensitivity analysis of the second DII quartile. The highest OR was observed after excluding the Aghaei et al. study, whereas the smallest OR was observed after omitting the Dabagh et al. study. Despite these variations, the pooled OR remained non-significant, suggesting that the overall results were relatively stable and were not strongly influenced by the exclusion of specific studies. **(B)** Sensitivity analysis of the third DII quartile. The highest OR occurred after excluding the study by Xu et al., whereas the lowest OR was noted after omitting the study by Dabagh et al. Despite these fluctuations, the pooled OR remained non-significant. **(C)** Sensitivity analysis of the fourth DII quartile. The greatest OR was observed after excluding the study by Liu et al., with the lowest OR following the omission of the study by Dabagh et al. Similar to the previous quartiles, the pooled OR remained non-significant after excluding these studies. **(D)** Sensitivity analysis of studies with continuous DII data. The highest OR was observed after excluding the study by Xu et al., whereas the lowest OR occurred after omitting the study by Lu et al. The pooled OR remained non-significant following the exclusion of these studies, indicating the robustness of the overall findings.

Heterogeneity was observed among studies (*p* < 0.001, I^2^ = 73.57%). The findings indicated that the effects of the DII on infertility in individuals in the second quartile were not statistically significant (OR, 1.25; 95% CI: 0.87–1.81) (Figure 2A). Subsequently, subgroup and sensitivity analyses were conducted. In the gender subgroup, homogeneity was observed for male individuals (OR: 4.17, 95% CI: 2.01–8.64), suggesting that an anti-inflammatory diet in males increased infertility odds by 4.17-fold. No statistically significant relationships were observed among the other subgroups (Table 3). Sensitivity analysis revealed that the highest OR occurred upon exclusion of the Aghaei et al. study (OR: 1.40, 95% CI: 0.95–2.05) (27), whereas the lowest OR was observed following the omission of the Dabagh et al. study (OR: 1.11, 95% CI: 0.79–1.56) (28). Despite these variations, the pooled OR remained non-significant after exclusion of these studies (Figure 3A). This evidence indicates that the overall result is relatively stable and that the findings are not strongly influenced by the exclusion of specific studies.

**Table 3:** Odds ratios (OR) and heterogeneity (I^2^) for subgroups by quartile.

Quartile	Subgroups		No. of studies	OR [95% CI]	I^2^%
Second quartile	County	Iran	4	1.49 [0.65–3.44]	85.66
		USA	4	1.25 [0.98–1.59]	0
	Study design	Case–control	1	0.60 [0.35–1.03]	0
		Cross-sectional	7	1.40 [0.95–2.05]	72.53
	Sex	Female	6	1.00 [0.76–1.33]	55.83
		Male	2	4.17 [2.01–8.64]	0
Third quartile	Country	China	1	1.05 [0.76–1.45]	-
		Iran	4	1.15 [0.91–1.45]	1.10
		USA	4	1.02 [0.82–1.26]	0
	Study design	Case–control	2	1.09 [0.82–1.43]	0
		Cross-sectional	7	1.06 [0.90–1.25]	0
	Sex	Female	6	1.04 [0.89–1.22]	0
		Male	3	1.32 [0.83–2.10]	27.96
Fourth quartile	Country	China	1	0.86 [0.58–1.27]	-
		Iran	4	1.77 [1.57–2.00]	0
		USA	4	1.70 [1.35–2.13]	0
	Study design	Case–control	2	1.17 [0.60–2.28]	74.06
		Cross-sectional	7	1.76 [1.58–1.96]	0
	Sex	Female	6	1.74 [1.56–1.94]	0
		Male	3	1.52 [0.65–3.57]	69.96
Continuous	Country	USA	4	1.10 [1.05–1.15]	0
	Study design	Cross-sectional	4	1.10 [1.05–1.15]	0
	Sex	Female	4	1.10 [1.05–1.15]	0

Each group is compared with the first quartile.

### 3.2 Third quartile

Nine studies assessed the effects of the DII on infertility in individuals in the third quartile (*p* = 0.68, I^2^ = 0%). The findings indicated that the effects of the DII on infertility in this group were not significant (OR: 1.07, 95% CI: 0.93–1.23) (Figure 2B). A subgroup analysis was then performed, yet no significant relationship between the DII and infertility was observed (Table 3). In the sensitivity analysis, the greatest change in OR occurred after the omission of the study by Xu et al. (OR: 1.10, 95% CI: 0.95–1.28) (35), and the least OR occurred after omitting the study by Dabagh et al. (28) (OR: 1.05, 95% CI: 0.91–1.21) (Figure 3B). This result suggests that the overall result is stable, with the findings not significantly affected by the exclusion of individual studies.

### 3.3 Fourth quartile

Nine studies evaluated the effects of the DII on infertility in individuals in the fourth quartile. A positive association was observed between the DII score and infertility (*p* = 0.08, I^2^ = 43.31%). After applying a random-effects model, the findings showed that the effects of DII on infertility in this population were significant (OR: 1.61, 95% CI: 1.32–1.95) (Figure 2C). The results indicate that individuals adhering to a pro-inflammatory diet showed 61% higher odds of infertility than those who did not follow such a diet. The subgroup analysis demonstrated significant associations between the DII and infertility across different populations and sexes. In both the Iranian and US populations, individuals adhering to a pro-inflammatory diet exhibited 77 and 70% higher odds of infertility, respectively, than those not following a pro-inflammatory diet. Additionally, female participants had 74% higher odds of infertility following a pro-inflammatory diet. Subgroup analysis according to the study design indicated that cross-sectional studies reported a 76% increase in the odds of infertility among individuals with a pro-inflammatory diet (Table 3). Finally, a sensitivity analysis revealed that the greatest OR was observed after excluding the study by Liu et al. (OR: 1.76, 95% CI: 1.58–1.95) (35), with the lowest OR following the omission of the study by Dabagh et al. (28) study (OR: 1.57, 95% CI: 1.29–1.91). The pooled OR remained non-significant after excluding these studies, and the results remained consistent (Figure 3C).

### 3.4 Continuous data

After evaluating the effects of continuous DII data on infertility in four studies, it was concluded that the DII had a positive impact on the prevalence of infertility (*p* = 0.66, I^2^ = 0%). Specifically, it was found that a one-unit increase in the DII increased the likelihood of infertility by 1.1 times (OR: 1.10, 95% CI: 1.05–1.15) (Figure 2D). The subgroup analysis showed consistent results across different categories. In the US population, among female participants, and in cross-sectional studies, all indicated a 10% increase in infertility odds for a one-unit increase in the DII. These findings suggest that the effect of DII on infertility is stable and is not influenced by study design, geographic location, or sex (Table 3). Sensitivity analysis showed that the highest OR was observed after excluding the study by Xu et al. (OR: 1.11, 95% CI: 1.05–1.17), while the lowest OR occurred after omitting the study by Lu et al. (OR: 1.09, 95% CI: 1.03–1.14). The pooled OR remained non-significant following exclusion of these studies, confirming the stability of the overall findings (Figure 3D).

### 3.5 Publication Bias

**Figure 4:**
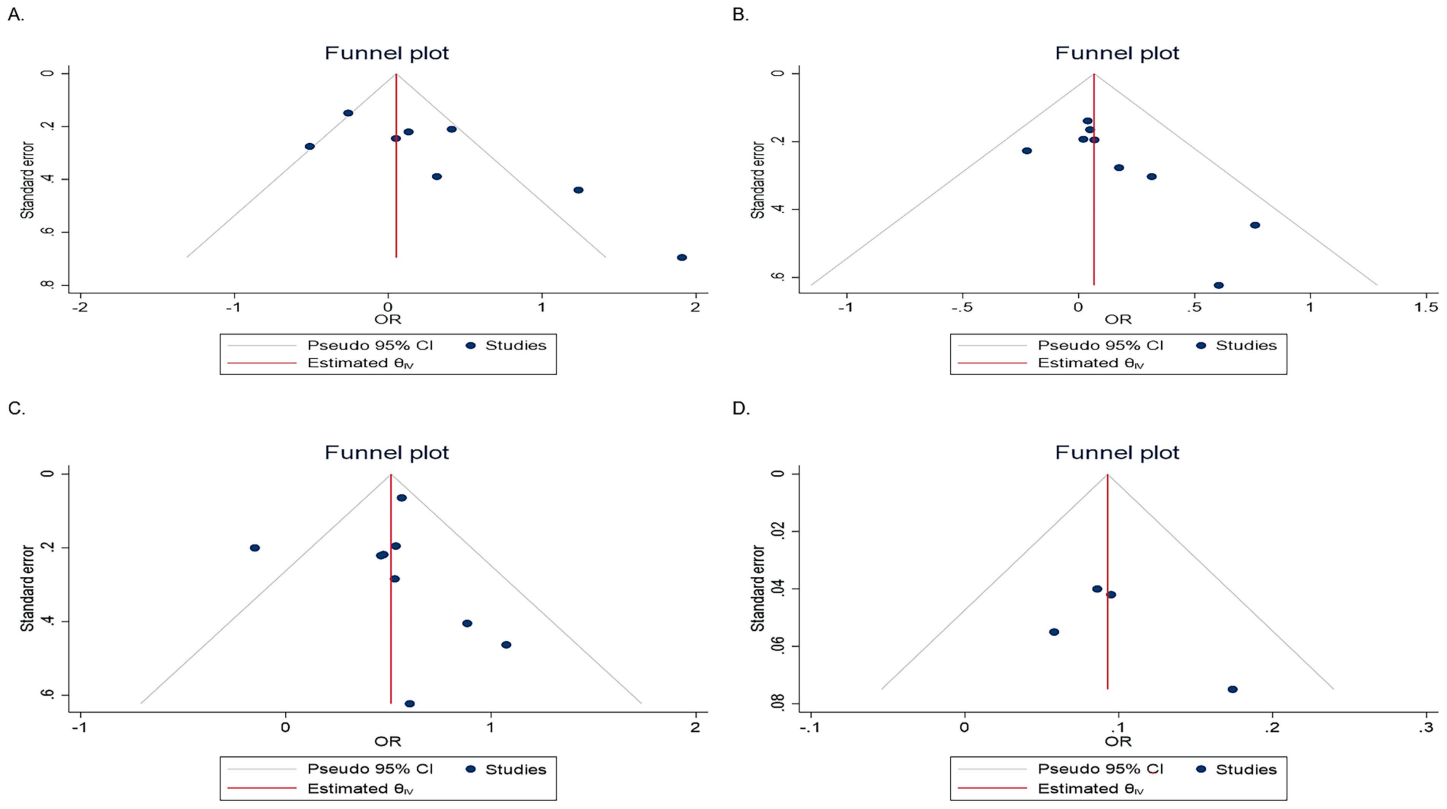
Funnel plots showing evidence of publication bias. **(A)** Second quartile, **(B)** third quartile, **(C)** fourth quartile, and **(D)** continuous data.

To assess the presence of publication bias, funnel plot analysis (Figure 4) and the Egger’s test were conducted. The Egger test results showed no significant evidence of publication bias for the third quartile (*p* = 0.15), fourth quartile (*p* = 0.43), or continuous data (*p* = 0.53) in the included studies. However, potential evidence of publication bias was observed in the second quartile (*p* = 0.02). Consequently, a trim-and-fill analysis was conducted to evaluate the potential impact of the second quartile on the pooled OR. The trim-and-fill analysis revealed that the pooled OR for the second quartile did not undergo significant changes following this adjustment.

## 4 Discussion

The effects of the DII on infertility were assessed across different quartiles. It was shown that there is an association between an anti-inflammatory diet and infertility in men. Furthermore, individuals adhering to a pro-inflammatory diet had higher odds of infertility compared to those not following such a diet. Increased odds of infertility were also observed in both the Iranian and US populations, as well as among female participants following a pro-inflammatory diet. Additionally, this study revealed a 10% increase in infertility odds per unit increase in DII.

Consistent with our findings, the National Health and Nutrition Examination Survey data revealed that higher DII scores were correlated with increased infertility risk. In this study, the OR for infertility was approximately two times greater for those on a pro-inflammatory diet than for those on an anti-inflammatory diet (32). In another related study, 4,437 female participants were assessed to determine the association between the DII and infertility in women, showing that participants with a pro-inflammatory diet had an 86% greater chance of infertility (31), which is in agreement with our findings. In accordance with our findings, other studies have found that adherence to an anti-inflammatory diet is positively associated with better sperm motility, count, and morphology, all of which are factors contributing to fertility (38–41). In contrast to these findings, Liu et al. conducted a study on 1,130 male participants and reported no significant relationship between the DII scores and asthenozoospermia risk (29), which is the leading cause of infertility. However, another study demonstrated a positive association between the DII scores and the risk of asthenozoospermia (42). This inconsistency may have been because of different dietary habits, sample sizes, and population characteristics.

As dietary inflammatory potential escalates, which correlates with an increase in DII scores, it can significantly elevate inflammatory markers, leading to systemic inflammation. This inflammation can affect fertility by decreasing sperm quality in men, adversely affecting the cervix, uterus, and placenta, and also increasing the risk of endometriosis and polycystic ovary syndrome (43–47). In addition, it has been observed that a pro-inflammatory diet can cause obesity and increase the risk of insulin resistance. Previous studies have shown that the presence of insulin resistance can cause pregnancy abnormalities and polycystic ovary syndrome. Elevated insulin levels during insulin resistance can disrupt spermatogenesis and reduce male fertility. Studies have indicated that individuals with polycystic ovary syndrome exhibit elevated levels of inflammatory markers such as TNF, IL-6, CRP, IL-18, IL-1β, and white blood cell counts (48–51). Endometriosis can impair fertility through its effects on gametes, embryos, fallopian tubes, and endometrium (52). These markers can cause inflammation, which disrupts endometrial function and hinders the decidualization of endometrial stromal cells (16).

Multiple studies have demonstrated that diet can change the gut microbiota (53, 54), which leads to inflammatory diseases (55, 56). Gut microbiota changes may affect infertility through inflammatory responses, which are induced by endotoxins or an increase in oxidative stress levels, damaging the DNA and leading to a reduction in sperm motility (57–60).

In men, higher levels of IL-1, IL-6, and TNF were detected in those with infertility (61). This high concentration of inflammatory markers can affect the prostate, which, in turn, affects sperm and fertility (62). An anti-inflammatory diet may indirectly enhance fertility by reducing adipose tissue and body weight (63). While the precise mechanisms through which an anti-inflammatory diet influences infertility are unclear, it has been suggested that such a diet may benefit reproductive health by improving its function (64).

As mentioned above, inflammation has many negative effects on an individual’s overall health. Shifting to a diet with anti-inflammatory characteristics not only improves fertility but also contributes to overall health improvement. However, our findings suggest that in males, a 4.17-fold increase in the odds of infertility is associated with an anti-inflammatory diet. While this information should be generalized with caution because, in the mentioned subgroup analysis, only two studies were included, it also highlights the complexity of the relationship between dietary patterns, inflammatory responses, and fertility. Nonetheless, integrating an anti-inflammatory dietary pattern could be considered as a way to treat and prevent infertility, thereby alleviating the burden of infertility in countries seeking to boost their populations.

### 4.1 Strengths and limitations

One of the strengths of this study is that it is one of the first meta-analyses to observe an association between the DII and infertility. Second, we conducted a comprehensive search of all important international medical databases, and all articles that investigated the DII and infertility were included in this study. This study had some limitations, which are as follows: There was heterogeneity seen in the second and third quartiles. To address this issue, we included the adjusted effect size and variables to exclude factors that may result in heterogeneity. Furthermore, subgroup analyses were conducted to explore the potential sources of heterogeneity across studies.

## 5 Conclusion

This meta-analysis demonstrated a significant relationship between the DII score and infertility risk, providing a significant association between anti-inflammatory diets and infertility in men and increasing infertility risk among individuals on a pro-inflammatory diet, particularly in both the Iranian and US populations, as well as among female participants following a pro-inflammatory diet. Additionally, each one-unit increment in the DII score was associated with a 10% increase in infertility odds. Our findings provide insight into how dietary patterns might impact reproductive health and underscore the importance of considering inflammation-related dietary factors in infertility prevention and treatment strategies. This study has important implications for public health, suggesting that dietary modifications targeting inflammation may offer cost-effective interventions for infertility management. However, because all the articles included in this study were observational, generalizing the results should be performed with caution. Further randomized controlled trials are necessary to elucidate a more profound knowledge of the relationship between the DII and infertility.

## Data Availability

The raw data supporting the conclusions of this article will be made available by the authors, without undue reservation.
